# Fighting abuse with prescription tracking: mandatory drug monitoring and intimate partner violence

**DOI:** 10.1007/s00148-025-01111-5

**Published:** 2025-06-28

**Authors:** Dhaval Dave, Bilge Erten, David Hummel, Pinar Keskin, Shuo Zhang

**Affiliations:** 1 Department of Economics, Bentley University, 175 Forest Street, Waltham, MA 02452 USA; 2 Department of Economics, Northeastern University, 43 Leon Street, 312A Lake Hall, Boston, MA 02115 USA; 3 Department of Economics, Wellesley College, Pendleton East, 106 Central Street, Wellesley, MA 02481 USA

**Keywords:** Prescription monitoring, Opioid misuse, Intimate partner violence, Domestic abuse, Health policy, United States, I18, J12, J16

## Abstract

The opioid crisis generates broader societal harms beyond direct health and economic effects, impacting non-users through adverse spillovers on children, families, and communities. We study the spillover effects of a supply-side policy aimed at reducing overprescription of opioids on women’s well-being by examining its effects on intimate partner violence (IPV) in the United States. Using administrative data on incidents reported to law enforcement, in conjunction with quasi-experimental variation in the adoption of stringent mandatory-access prescription drug monitoring programs, we find that these policies generate a downstream benefit for women by significantly reducing their overall exposure to IPV and IPV-involved injuries by 9 to 10%. The strongest effects are experienced by groups with higher rates of opioid consumption at baseline, including non-Hispanic Whites. Our findings also show a significant increase in heroin-involved IPV incidents, suggesting substitution into illicit drug consumption. However, since heroin-related IPV accounts for less than 1% of all incidents, its increase among highly opioid-dependent individuals does not offset the overall decline in total IPV incidents in affected states. Our results highlight the need to identify high-risk groups prone to switching to illicit opioids and to address this risk through evidence-based policies.

**Supplementary Information:**

The online version contains supplementary material available at 10.1007/s00148-025-01111-5.

## Introduction

The ongoing opioid epidemic in the U.S. has had profound effects on public health and engendered far-reaching consequences for families and communities. Since 1999, more than 700,000 individuals have died from drug overdoses involving opioids.^1^ The annual economic burden of the opioid crisis, which amounted to over $\$$1 trillion in 2017, reached $\$$1.5 trillion in 2020 as misuse and overdose continued to rise sharply through the COVID-19 pandemic.^2^ As staggering as these magnitudes are, they are likely under-stated since these estimates are largely limited to directly attributable costs imposed by opioid misusers on the healthcare system, criminal justice system (i.e., from drug-related crime), and productivity (i.e., from premature deaths and incarceration).^3^ Broader societal harms generated by the opioid crisis, however, extend well beyond these direct health and economic effects and encompass impacts among non-users through adverse spillovers on children, families, and communities. Recent evidence, for instance, has underscored just such wide-ranging impacts; studies have linked the opioid crisis with worse infant health (Gihleb et al. 2020; Ziedan and Kaestner 2020), child abuse and maltreatment (Duane et al. 2019; Gihleb et al. 2022; Evans et al. 2022), deteriorating economic conditions and labor market prospects (Harris et al. 2020; Cho et al. 2021; Beheshti 2023; Aliprantis et al. 2023), and property and violent criminal offenses (Dave et al. 2021; Maclean et al. 2022; Mallatt 2022).

A key implication of such downstream effects associated with the opioid epidemic is that interventions targeted at curbing opioid abuse—either from the demand-side or the supply-side—may end up further impacting populations and outcomes beyond those that were targeted or intended. Guiding effective intervention strategies to not only lessen opioid misuse but also contain its adverse downstream effects on non-users, children, and families thus requires a comprehensive accounting of how these policies are affecting a broad range of outcomes and populations.

**Fig. 1:**
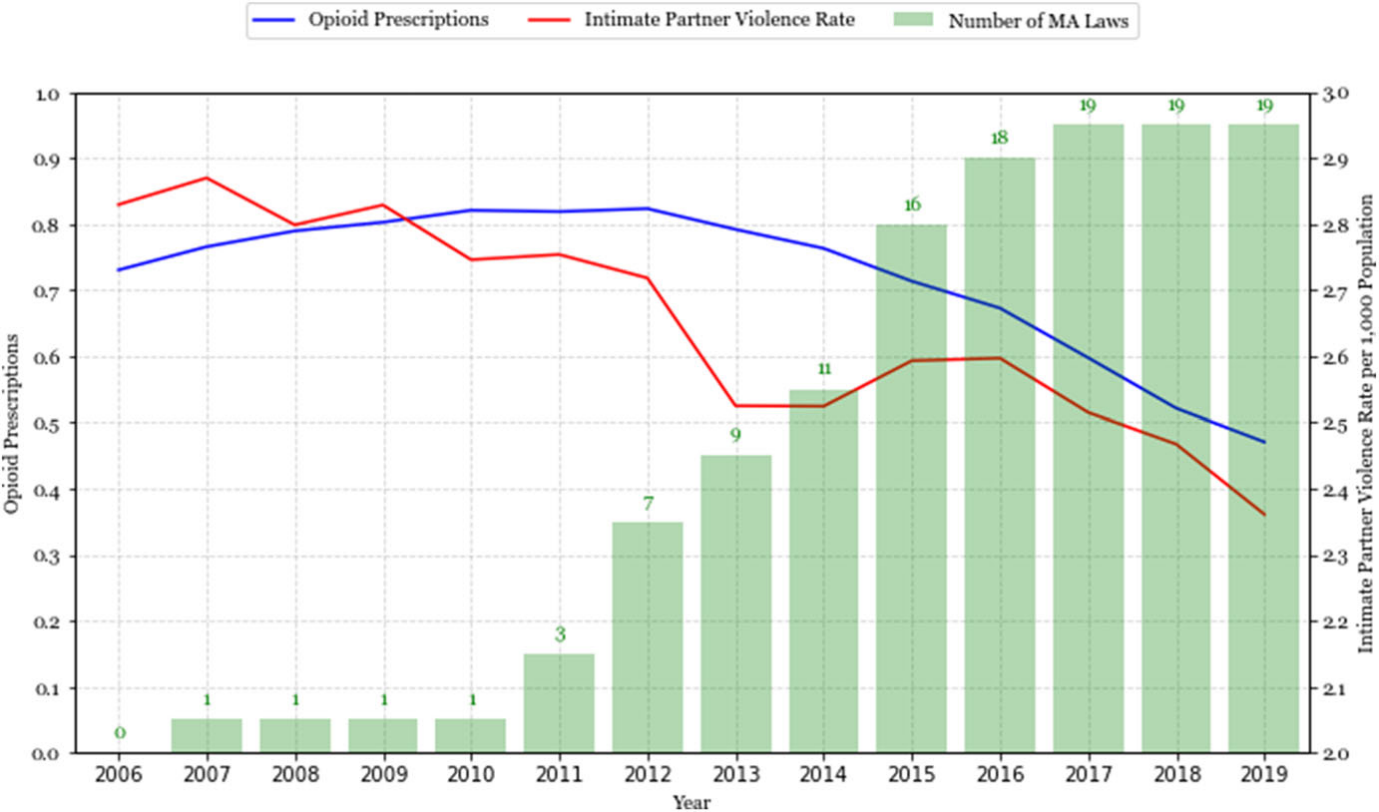
Opioid prescriptions per capita, IPV rate, and mandatory-access PDMPs. *Note:* In this figure, the blue line shows annual opioid prescriptions per capita as reported by the CDC, and the red line shows the intimate partner violence rate per 1000 people, calculated using data from the 2006–2019 NIBRS. The green bars display the number of states that implemented mandatory-access PDMPs in a given year. Opioid prescriptions per capita refers to the population-weighted median number of prescriptions each year. The intimate partner violence rate is the yearly average number of incidents per 1000 population

In response to the first wave of the opioid epidemic (spanning until about 2010; see Fig. 1), which involved a surge in the prescribing of opioids and overdose deaths involving these prescription (Rx) opioids, an increasingly popular policy tool adopted by states was the implementation of prescription drug monitoring programs (PDMPs). PDMPs are state-run electronic databases that track the prescribing and dispensing of controlled substances, providing critical information on the patient’s prescribing history to healthcare providers (i.e., physicians, pharmacies). With the overprescription of opioids being a significant catalyst for the advent of the public health crisis, PDMPs target inappropriate prescribing for patients who may have a history of or are at risk of opioid abuse, and importantly identify patients who may be “doctor shopping,” that is obtaining opioid prescriptions from multiple providers and pharmacies for their own use or for diversion into illicit markets.

A large literature has evaluated immediate and direct impacts of PDMPs on targeted outcomes such as opioid prescriptions, sales, misuse, and overdose mortality (Buchmueller and Carey 2018; Grecu et al. 2019; Kaestner and Ziedan 2023; Maclean et al. 2022; Wen et al. 2019). A consistent finding to emerge from these studies is that earlier versions of the PDMP, which were voluntary and did not mandate registration and access, had little to no effect on prescribing or measures of misuse; however, modern PDMP designs, which mandate provider access prior to prescribing opioids, have been found to be highly effective in reducing opioid misuse and overdose mortality. Complementing these findings is some emerging evidence that the impacts of these policies are broader and not confined to just constraining the supply of prescription opioids. Kim (2021) finds, for instance, that mandatory-access PDMPs, while reducing overdose mortality associated with Rx opioids, generate an unintended adverse consequence and lead to more deaths involving heroin overdose as potential users substitute from Rx opioids to illicit opioids. Counteracting such negative spillover effects, studies have also uncovered beneficial downstream effects on communities and families. Dave et al. (2021) find that mandatory-access PDMPs impart a heretofore unidentified benefit for communities in the form of lower criminal activity, particularly aggravated assault, burglary, and homicides among young adults. Introduction of mandatory provisions to PDMPs has also been found to benefit children by reducing cases of child removals associated with maltreatment and reducing admissions into the foster care system (Gihleb et al. 2022).

Our study contributes to this emerging evidence base on how PDMPs specifically—and supply-side interventions targeted at the healthcare system more broadly—can generate downstream effects on non-targeted outcomes and populations. We draw focus, in particular, on spillovers on women’s well-being by exploring effects on intimate partner violence experienced by women—an outcome that has largely remained unexplored in the opioid policy literature. Intimate partner violence (IPV) is the most prevalent form of violence perpetrated against women, with 47.3% of women in the U.S. reporting being a victim of IPV at some point in their lifetime (Smith et al. 2022).^4^ Several pathways can mediate a possible causal link from PDMPs to women’s experience of IPV. By shifting use and misuse of opioids among their current or former partners, these policies could impact the risk of IPV perpetration through the drugs’ direct pharmacological effects, that is, by affecting users’ aggressive tendencies, impulse control, and emotional dysregulation.^5^ Indirect economic effects on employment and earnings and on intra-household conflict may further mediate impacts on women’s exposure to IPV. Moreover, we note that these channels can impact women’s exposure to IPV both through effects on potential perpetrators as well as through “own effects” by affecting female opioid users’ risk of victimization.

We leverage administrative information from incident-based reports to law enforcement agencies in 31 states (derived from the National Incident Based Reporting System (NIBRS)), combined with spatio-temporal variation in the adoption of PDMP policies, to derive plausibly causal effects. We utilize data from 2006 to 2019, a period that spanned all three waves of the opioid epidemic and witnessed considerable state activity on PDMP regulations (i.e., 19 states enacted PDMP mandates over this period; see Supplementary Material Table A1). Analyses are based on a generalized difference-in-differences approach, supplemented with newly developed estimators that account for heterogeneous treatment effects across treated units and over time.

Our study documents several key findings. First, we find that the enactment of mandatory-access PDMP provisions resulted in a significant decline, on the order of about 9%, in female-reported IPV incidents, over a post-treatment observation window of 6 years for the average treated state. Second, there are strong dynamics at play with these spillover effects materializing with a lag of about four years post-adoption. Third, we also document a significant 9.5% decline in the incident reports of injuries related to IPV, indicating that the observed decline in the IPV rate is not driven by a shift in reporting behaviors. Fourth, while the decline in women’s exposure to IPV points to a beneficial spillover of constraining access to Rx opioids, this decline is partly offset by a small but potentially meaningful uptick of IPV incidents committed by perpetrators suspected of heroin use. This is consistent with potential substitution from Rx opioids to illicit opioids among a subset of users who saw their access to Rx opioids restricted by the PDMP regulations. Another implication of this result is that the overall decline in women’s exposure to IPV that we find is at least partly driven by a decreased risk of IPV perpetration and cannot wholly be explained by a lower risk of victimization among female opioid users. Fifth, we do not find any statistically or economically significant effects for voluntary PDMP programs on any of the IPV outcomes, which is validating given that prior work also did not find such programs to have any substantial “first-stage” impacts on opioid use and misuse. Finally, heterogeneity analyses uncover that the largest benefits in terms of an overall net decline in IPV accrue to non-Hispanic white and younger adults; the corollary uptick in exposure to IPV with heroin involvement is also largest for this subpopulation. Event-study analyses, including those generated from standard two-way fixed effects models, as well as various alternate estimators, support a causal interpretation of these findings.

In providing some of the first comprehensive evidence on the potential spillover effects of PDMPs on women’s exposure to IPV, our study makes several contributions. First, we contribute to the growing literature on risk factors that affect the incidence of IPV. Most of these studies have focused on economic shocks or other policies that may impact women’s bargaining power by documenting the effects of cash transfers (Bobonis et al. 2013; Angelucci 2008), labor market shocks (Aizer 2010; Anderberg et al. 2016), education (Erten and Keskin 2018), divorce laws (Stevenson and Wolfers 2006), and trade shocks (Erten and Keskin 2024) on the risk of IPV.

Although substance use has long been identified as a significant proximate and distal risk factor for IPV perpetration, much of the focus in this literature has centered on alcohol and much of the work is correlational in nature.^6^ Exceptions to this are two recent studies that analyze the effects of supply-side opioid policies on IPV risk. First, Dave et al. (2023) focus on a national intervention—the 2010 reformulation of OxyContin into an abuse-deterrent form—and leverage spatial variation in pre-intervention opioid prescriptions to identify a causal response. They find significant reductions in women’s exposure to IPV as a result of this reformulation. The findings from our study complement this work by shifting the focus to another increasingly popular supply-side restriction with marked differences. In contrast to the reformulation shock, which eliminated the supply of an abuse-prone form of a widely misused opioid, PDMPs seek to indirectly restrict access to all prescription opioids and do so specifically for patients who are at a higher risk of inappropriate use by influencing providers’ prescribing behaviors. Moreover, while the OxyContin reformulation was a nationwide supply shock, mandated access PDMPs have not been universally adopted, consequently generating considerable regional variation and scope for further policy action.

In the only other work to explore the impact of PDMPs on domestic violence, a recent paper by Barbos and Sun (2021) finds a significant decrease in simple assaults between intimate partners on the order of about 7%. Barbos and Sun (2021) focus their analyses on domestic violence perpetrated by older adult offenders ages 35–64 at the time of the incident; in bypassing younger adult offenders, their analyses select out an important population for whom both the risk of opioid abuse as well as IPV perpetration (and by corollary, the risk of IPV exposure among younger adult victims, due to assortative mating) is substantially higher. Widening the lens to both younger and older victims and offenders is revealed to be an important distinction in understanding the spillover effects of these policies. Additionally, we pay particular attention to dynamics in the policy responses. Notably, in light of important lags that have been identified in the policy literature on PDMPs (see, for instance, Grecu et al. 2019), we implement recently introduced difference-in-differences methods designed to curb potential biases in two-way fixed effects methods that arise from dynamic and spatial heterogeneity in treatment effects within a staggered adoption setting. By combining our estimates of the impacts on IPV exposure, in conjunction with the robust body of evidence that has established strong “first-stage” effects on Rx opioid use and misuse, our study informs the causal role of the opioid epidemic in driving changes in IPV.

Second, in studying a supply-side intervention focused on one part of the opioid market—namely, opioids prescribed within the health care setting—we are able to draw focus on potential substitution effects into illicit opioids (i.e., heroin) and the implications of this substitution for women’s exposure to IPV. As our analyses span the evolution of the opioid epidemic across all three waves—from the run-up in overdose mortality related to Rx opioids to subsequently shifting to heroin and further moving towards synthetic opioids including fentanyl—we emphasize dynamics in how downstream effects potentially materialize and play out. Third, given the substantial economic burden of IPV against women, amounting to $\$$11.6 billion annually or almost $\$$140,000 in lifetime per-victim costs (in 2023 dollars), if there are spillovers of opioid policies on IPV, they are likely to be of an order of cost magnitude that is economically significant.^7^ Not accounting for these costs could underestimate the societal burden of the opioid epidemic and skew the cost-benefit calculus of policy interventions. Our study draws on the IPV estimates to inform how incorporating broader effects on IPV experienced by women potentially adds to the cost burden.

The remainder of the paper is organized as follows. Section 2 provides some policy background and details the data used in our analysis. Section 3 presents our research design and estimation strategy, followed by a discussion of the results in Sect. 4. Finally, Sect. 5 concludes with policy and welfare implications.

## Background and data

### Introduction of prescription drug monitoring programs (PDMPs)

The opioid crisis has been exacerbated by physicians’ prescribing practices (Kolodny et al. 2015). The introduction of new drugs, such as OxyContin in 1996, coupled with aggressive pharmaceutical marketing throughout the 1990s, played a major role in convincing the medical community that opioids were a safe and effective pain management solution, downplaying the addiction risks (National Academies of Sciences, Engineering, and Medicine and others 2017; Humphreys et al. 2022). At the same time, there was a growing concern about inadequate pain treatment, which led to the adoption of more aggressive pain management protocols. Consequently, state medical boards relaxed the regulations governing the prescription of opioid analgesics for chronic noncancer pain. This combination of aggressive marketing, the availability of potent new opioids, and eased prescription rules led to the overprescription of opioids, paving the way for widespread misuse and the resulting public health crisis. From 1991 to 2010, the number of opioid prescriptions in the US rose sharply from 76 to 250 million (Volkow 2014).

Part of what enabled such widescale overprescribing is the practice known as “doctor shopping,” where patients would obtain prescriptions from multiple doctors without knowing about prescriptions from other practitioners. Doctor shopping is not only for personal use, but is a significant source of supply for dealers (Inciardi et al. 2009). To reduce doctor shopping and effectively address the problem of overprescribing, states began implementing prescription drug monitoring programs (PDMPs), which are state-run electronic databases that track the dispensing of controlled substances across healthcare providers. The primary role of PDMPs is to identify possible patterns of medication misuse, especially regarding opioids. With the ability to track prescriptions and dispensing of controlled substances, healthcare providers can review a patient’s prescription history before prescribing to them, making it difficult for patients to acquire opioids from different sources.

Since their initial introduction in the late 1990s, PDMPs have evolved significantly. Earlier versions of PDMPs were voluntary, which made it optional for healthcare providers to access the database. However, these voluntary systems had a limited to no effect on controlling prescription drug abuse (Brady et al. 2014; Jena et al. 2014; Meara et al. 2016; Grecu et al. 2019). Recognizing this shortfall, many states have improved and modernized their programs by instituting universal registration and mandatory-access provisions, requiring healthcare providers to register with and query the PDMP before prescribing controlled substances or face disciplinary action from the state’s appropriate licensing board (Sacarny et al. 2023). This structure ensures a more consistent and reliable approach to monitoring and preventing prescription drug misuse. Audits from individual states have demonstrated that mandatory-access PDMPs increase utilization and query rates (Grecu et al. 2019; Dave et al. 2021). Empirical studies have documented that these more stringent programs have reduced prescription opioid misuse. Specifically, mandatory-access PDMPs have decreased opioid misuse among Medicare Part D participants (Buchmueller and Carey 2018) and have also reduced opioid misuse and opioid-related deaths among the general adult population (Grecu et al. 2019). However, recent studies have also shown that while mandatory-access PDMPs reduce prescription opioid deaths, this decrease could be offset by a large increase in illegal opioid deaths, including heroin (Kim 2021).

Supply-side interventions such as PDMP regulations and their downstream effects on women’s exposure to IPV are connected through many links in the causal chain. First, state reports have shown strong dynamics in PDMP registration and query rates by physicians subsequent to the adoption of a statutory mandate. While these mandates have been found to be highly effective in raising use rates by physicians, reports (i.e., the early adopter states KY, OH, NY, TN) have shown substantially stronger effects over time, taking as much 3–4 years and longer for maximal impact (PEW 2016; Strickler et al. 2019; TTAC 2020). These dynamics have generally been attributed to barriers to PDMP use, such as access difficulties and “down” systems, lack of time, inability to delegate access, lack of knowledge or awareness, lack of integration with electronic health records and into clinical workflow, and minimal training and guidance to assist users (Haffajee et al. 2015; Martin et al. 2021). As a result, there was a steep learning curve as physician practice patterns evolved with accumulating experience with PDMP use.

Second, consistent with these lags in the “first-stage” effects on query and use rates, most of the literature that has assessed impacts on measures of Rx drug misuse, doctor shopping, and opioid prescribing behaviors also find significant lags and strong dynamics. Studies on supply-side shocks/policies that restrict access to Rx opioids (such as PDMP regulations, OxyContin reformulation) further point to potential stockpiling of supplies and/or alternate sources of supply in the short run as additional drivers of lagged responses (Dave et al. 2021; Powell and Pacula 2021; Park and Powell 2021; Beheshti and Kim 2022; Gihleb et al. 2022; Dave et al. 2023). These initial lags in the first-order causal links on PDMP use, and on measures of opioid prescribing and misuse, are further compounded by lags in the second-order causal links shaping how current and cumulative substance use maps into exposure to IPV (both from a higher risk of victimization and perpetration). Finally, given that we rely on administrative reports of IPV to law enforcement by female victims, it is important to note that not all forms of IPV get reported and not all victims report to law enforcement; it may take some time for IPV exposure to reach a threshold (for instance, either through cumulative exposure or being exposed to a particularly severe form of IPV) for victims to file a report. Each of these links in the causal chain adds to the delay in which potential effects may materialize.

We use data from Evans et al. (2022) to determine and cross-reference the adoption dates of mandatory-access PDMPs across states. Supplementary Material Table A1 provides a list of the years in which states implemented mandatory-access PDMPs. In 2007, Nevada pioneered the inclusion of a “must-access” provision in its PDMP, mandating providers to both report all prescriptions and consult the PDMP to review a patient’s prescription history before prescribing controlled substances. Figure 1 shows that several other states implemented mandatory-access PDMPs subsequently. Oklahoma followed with its own must-access provision in 2010, and Ohio did so in 2011. The adoption of mandatory-access PDMPs increased over time in 2010s and reached a peak of 19 states by the end of our sample period, 2019 (Fig. 1). At the same time, opioid prescriptions increased from 0.72 to 0.81 per person from 2006 to 2010, remained steady from 2010 to 2012, and then declined to 0.47 by 2019.

### IPV data

We use police-reported intimate partner violence (IPV) incidents recorded in the National Incident-Based Reporting System (NIBRS) from 2006 to 2019. This system, managed by the Federal Bureau of Investigation (FBI), collects data on crimes reported by police agencies at the incident level. The data includes details such as the date and location of the incident, characteristics of the victims and offenders, and the types of crimes recorded in the incident. Specifically, each report in the NIBRS contains detailed information regarding the characteristics of the victim and the offender, such as age, gender, race, and ethnicity. Importantly, the NIBRS also has information on the relationships between victims and offenders, and for offenders, the NIBRS also reports whether they were suspected of using substances, including heroin. At the incident level, the data also includes whether the incident resulted in an injury or an arrest. Compared to individual survey data, this dataset has several advantages: it is less reliant on self-reports, has been gathered over an extended period, and allows us to determine whether an offender was suspected of using opioids.

Our analysis focuses on IPV incidents experienced by female victims, where the relationships with the offenders include spouses, common-law spouses, boyfriends/girlfriends, homosexual partners, ex-spouses, and ex-boyfriends/girlfriend. The IPV incidents include aggravated assaults, simple assaults, forced sex, and intimidation. Our primary outcome measure is annual IPV rate per 1000 population at the county level. We use a balanced panel of county-level data from 2006 to 2019 including more than 9000 reporting law enforcement agencies.^8^ Our additional outcome measures include opioid-involved IPV rate per 1000 population, which is the rate of IPV incidents where the police suspected that the offender was using opioids at the time of the incident; the injury rate per 1000 population, and the arrest rate per 1000 population, all of which are measured at the county level.

Supplementary Material Table A2 presents summary statistics for the variables used in our analysis. The annual average IPV incident rate was 2.6 per 1000 population at the county level from 2006 to 2019, with about half of these incidents resulting in an injury and arrests. Figure 1 displays a declining annual trend for the average IPV rate over this time period from about 2.8 per 1000 in 2006 to slightly less than 2.4 per 1000 in 2019.

### Data on covariates

We use several additional sources of data to account for time-varying county characteristics with potential to affect our outcomes of interest. First, we use demographic data from the Surveillance, Epidemiology, and End Results (SEER) Program, which collects data from the U.S. Census Bureau: the share of Black, White, and Hispanic populations, and share of population within different age brackets: 0–19, 20–24, 25–34, 35–44, 45–54, 55–64, and 65 or older at the county level. From the American Community Survey (ACS), we use data on the share of female adults at the county level. From the CDC, we use information on the rate of cancer deaths per 100,000 individuals to account for time-varying health conditions and pain prevalence at the county level. Finally, we use data from the Bureau of Labor Statistics (BLS) on average unemployment rate and the labor force participation rate at the county level to control for time-varying socioeconomic conditions at the county level. Additionally, in the NIBRS dataset, numerous agencies can report from a given country each year. To ensure data quality, we control for the number of agencies reporting any IPV incidents within each county and year, using incident data from the NIBRS, following previous studies (Freedman and Owens 2011; Thomas and Shihadeh 2013).

Furthermore, we control for initial county characteristics (measured in 2006) interacted with year fixed effects to isolate differences in baseline characteristics that could lead to differential outcomes over time. Specifically, we first use data from the American Community Survey to measure the initial share of population without any college education to account for exposure to labor-saving technological changes and the associated deaths of despair, which reflect a combination of negative social and economic outcomes that build up over time (Case and Deaton 2017, 2020). Second, we use data from the BLS on the share of employment in mining to account for the higher injury rates associated with underground mining, which increases opioid usage and mortality (Monnat 2018; Metcalf and Wang 2019).

Finally, we control for the following state policies: indicators for whether the state has a medical marijuana law, and whether the state has adopted the Medicaid expansion under the Affordable Care Act (ACA) in our baseline analysis. We incorporate additional state policies in robustness analyses. Summary statistics for these variables are provided in Supplementary Material Table A2.

## Empirical strategy

The primary objective of this paper is to identify the causal effect of must-access PDMPs on IPV outcomes. A simple correlation might suffer from significant endogeneity concerns and, as a result, cannot be interpreted as indicating causation. Such endogeneity concerns include omitted variable bias. For instance, states might have adopted must-access PDMPs due to unobserved factors that could be associated to both opioid prescriptions and partner abuse.

To obtain estimates that can be credibly interpreted as causal, we leverage the staggered rollout of must-access PDMPs from 2007 to 2019. Under a set of assumptions that we describe below, the quasi-experimental variation generated by the staggered PDMP rollout allows us to estimate the causal impact of these programs using a generalized difference-in-differences strategy. Specifically, the strategy compares the before-after difference in IPV outcomes between states where must-access PDMPs were introduced and states that did not change their PDMP status between the two periods.

For our baseline specification, we estimate the following dynamic two-way fixed-effect (TWFE) model:

1 $$\begin{aligned} Y_{cst} = \alpha _{c} + \delta _{t} + \sum _{\begin{array}{c} t=-\tau , t \ne -1 \end{array}}^{T} \beta _t Time_{t} * PDMP_{s} + \textbf{X}_{cst} + \epsilon _{cst} \end{aligned}$$

where $Y_{cst}$ represents an IPV outcome for county *c* in state *s* at year *t*. The county fixed effects, $\alpha _{c}$, absorb any unobserved time-invariant characteristics at the county level (e.g., gender norms on acceptability of violent behavior between partners). The period fixed effects, $\delta _{t}$, account for any shocks that may affect all counties at the same time. $PDMP_{s}$ is an indicator for treatment states where a must-access PDMP policy was introduced, and $Time_{t}$ are a set of time period indicators corresponding to the year since the policy implementation. The coefficients $\{\beta _0, \ldots , \beta _T\}$ identify dynamic treatment effects, $\beta _{-1}$ is the omitted category, and $\beta _{-\tau }, \ldots , \beta _{-2}$ estimate anticipation effects. $\textbf{X}_{cst}$ is a vector of covariates that vary across counties and over time composed of three terms: (i) demographic and socioeconomic covariates at the county level (including the percentage of female, White, Black, and Hispanic populations; the number of cancer deaths per 100,000 individuals; percentage of the population under age 19, between ages 20 and 24, 25 and 34, 35 and 44, 45 and 54, and 55 and 64; unemployment and labor force participation rates; the number of agencies reporting any IPV incidents within each county and year), (ii) state policies (including indicators for a medical marijuana law and whether the state had expanded ACA coverage), and (iii) initial county characteristics interacted with period indicators (including share of population without any college education and the share of employment in mining as discussed in the previous section). Adding interactions of these characteristics with the full set of period dummies allows their relationship with IPV rates to differ before and after the policy implementation. We weight all regressions by 2006 county population. We estimate Eq. 1 using ordinary least squares (OLS) and cluster standard errors at the state level to account for serial correlation in the error term within a state.

To the extent that, in the absence of the must-access PDMP rollout, the IPV rates across treated and control states would have evolved along parallel trends, and assuming state-level average treatment effects are homogeneous across treated states and over time, the coefficients of interest $\{\beta _0, \ldots , \beta _T\}$ identify the dynamic treatment effects on the treated of the introduction of must-access PDMPs on the IPV rate.

While TWFE regressions similar to Eq. 1 have been the benchmark models for staggered adoption research designs, they have been shown to yield consistent estimates only under strong assumptions about homogeneity in treatment effects (De Chaisemartin and D’Haultfoeuille 2020; Goodman-Bacon 2021; Sun and Abraham 2021; Borusyak et al. 2024). To ensure robustness of our findings, we also present the event study estimates using the robust estimators that produce consistent estimates under treatment effect heterogeneity. Specifically, we use estimators introduced in De Chaisemartin and D’Haultfoeuille (2020), Sun and Abraham (2021), and Borusyak et al. (2024).

Complementing the event-study specification, we estimate the average treatment effects of the must-access PDMPs on the IPV rate. We estimate the following specification to capture the short-run and medium-run effects of PDMPs:

2 $$\begin{aligned} \begin{aligned} Y_{cst}&= \alpha _{c} + \delta _{t} + \beta _1 SR_{POST_t} * PDMP_s + \beta _2 MR_{POST_t} * PDMP_s + \textbf{X}_{cst} + \epsilon _{cst} \end{aligned} \end{aligned}$$

where $SR_{POST_t}$ is an indicator taking the value of 1 if it is 0 to 3 years after the PDMP implementation in state *s*; $MR_{POST_t}$ is an indicator taking the value of 1 if it is 4 to 6 years after the PDMP implementation in state *s*. This specification also includes county fixed effects $\alpha _{cs}$, year fixed effects $\delta _{t}$, and a vector of covariates $\textbf{X}_{cst}$ as defined in Eq. 1. We weight all regressions by the 2006 county population. We estimate Eq. 2 using the robust estimator proposed by Borusyak et al. (2024) and cluster standard errors at the state level.

Our choice to analyze data at the county level, despite the policy being implemented at the state level, is motivated by the substantial heterogeneity within states. Factors such as population density, socioeconomic conditions, and geographic characteristics can vary significantly between counties, even within the same state. Conducting the analysis at the county level allows us to better capture these variations and control for localized confounding factors. This approach ensures that our estimates more accurately reflect the relationship between the policy and outcomes of interest. However, we acknowledge that county-level analysis introduces potential challenges, such as spatial spillover effects, which we address by estimating robust standard errors clustered at the state level.

## Results

### Dynamic treatment effects

**Fig. 2:**
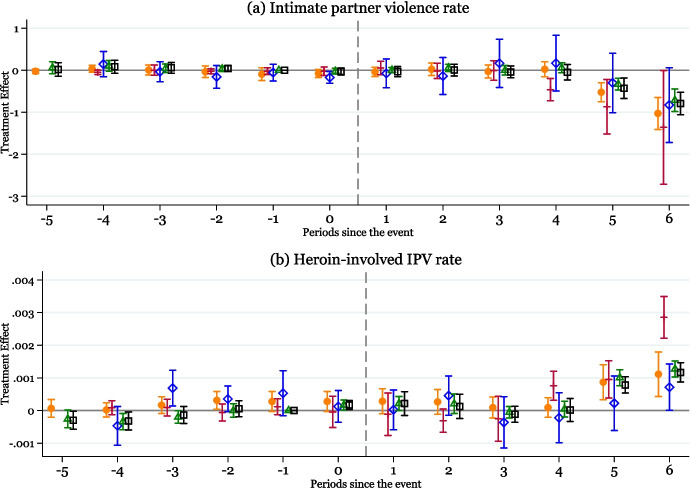
The effects of mandatory-access PDMPs on IPV rates over time. *Note:* Data are from the 2006–2019 NIBRS. Event-study plots show the response of IPV rate and heroin-involved IPV rate per 1000 population reported by female victims at the county level (*N*=12,487 county-years) to mandatory-access PDMP implementation. This figure overlays the event-study plots based on Eq. (1) using five different estimators: Borusyak, Jaravel, and Spiess (2024) (in orange with circular markers); De Chaisemartin and d’Haultfoeuille (2020) (in red with cross markers); Callaway and Sant’Anna (2021) (in blue with diamond markers); Sun and Abraham (2021) (in green with triangle markers); and a dynamic version of the TWFE model, Eq. (1), and estimated using OLS (in blue with black square markers). Each figure reports treatment effect estimates and 95% confidence intervals. Specifications include county and year fixed effects, county-level covariates (percent female, White, Black, Hispanic population; number of cancer deaths per 100,000 population; percent population under age 19, between 20 and 24, between 25 and 34, between 35 and 44, between 45 and 54, and between 55 and 64; unemployment and labor force participation rates, the number of agencies reporting any IPV incidents), initial county characteristics (share of population without any college education and the share of employment in mining) interacted with year fixed effects, and state-level policies (indicators for a medical marijuana law and ACA expansion). Standard errors are clustered at the state level

We start by presenting dynamic treatment effects of the mandatory-access PDMPs on IPV outcomes. Figure 2 shows the event-study estimates based on Eq. 1. In all figures, the treatment effects for the time period prior to the implementation of mandatory-access PDMPs are close to zero and exhibit no discernible trends across five different estimators, consistent with the common trends assumption. Panel a shows that the IPV rate initially shows no significant changes for a period of up to three years in states that implemented mandatory-access PDMPs after the implementation; however, a clear downward shift appears approximately three to four years post-implementation (depending on the estimator). The delayed response to mandatory PDMPs is expected for several reasons. First, it takes considerable time for the healthcare providers to comply with the new regulations by registering and consulting the PDMP prior to prescribing opioids as described in Sect. 2. Second, even under mandatory-access PDMPs, individuals may continue to have access to prescription opioids for misuse due to stockpiling and illicit drug markets. Given these considerations, delayed effects that compound over time are a common feature of results documented by previous studies on the effects of mandatory-access PDMPs (Dave et al. 2021; Powell and Pacula 2021; Park and Powell 2021; Beheshti and Kim 2022; Gihleb et al. 2022; Dave et al. 2023). Table 1 reports the average treatment effects estimated based on Eq. 2. These results show that in the 3 years post-implementation, there is no evidence of a significant change in the IPV rate in the short run. However, the medium-run estimate indicates a 9% annual decrease in IPV rate in the states that implemented mandatory-access PDMPs in the 4–6 years post-implementation compared to states that did not implement these programs.^9^

**Table 1:** The effects of mandatory-access PDMPs on IPV rates

Panel A: Impact of mandatory-access PDMPs on IPV rate and heroin-involved IPV rate		
	IPV rate	Heroin-involved IPV rate
	(1)	(2)
Short-run post-PDMP (0$\le$t$\le$3)	$-$0.0290	0.0002
	(0.0813)	(0.0002)
Medium-run post-PDMP (3<t$\le$6)	$-$0.2596**	0.0004**
	(0.1245)	(0.0002)
Observations	12,487	12,487
Pre-policy outcome mean	2.7980	0.0001
Panel B: Impact of mandatory-access PDMPs on injury and arrest rates		
	Injury rate	Arrest rate
	(1)	(2)
Short-run post-PDMP (0$\le$t$\le$3)	$-$0.0444	0.0023
	(0.0334)	(0.0505)
Medium-run post-PDMP (3<t$\le$6)	$-$0.1325**	$-$0.0161
	(0.0562)	(0.0697)
Observations	12,487	12,487
Pre-policy outcome mean	1.4014	1.4988

*Notes:* Data are from the 2006–2019 NIBRS. Analyses show the response of IPV rate, heroin-involved IPV rate, injury rate, and arrest rate per 1000 population reported by female victims at the county level (*N*=12,487 county-years) to mandatory-access PDMP implementation. Estimates are calculated using the Borusyak et al. (2024) method using the specification in Eq. 2. Specifications include county and year fixed effects, county-level covariates (percent female, White, Black, Hispanic population; number of cancer deaths per 100,000 population; percent population under age 19, between 20 and 24, between 25 and 34, between 35 and 44, between 45 and 54, and between 55 and 64; unemployment and labor force participation rates, the number of agencies reporting any IPV incidents), initial county characteristics (share of population without any college education and the share of employment in mining) interacted with year fixed effects, and state-level policies (indicators for a medical marijuana law and ACA expansion). Standard errors in parentheses are clustered at the state level. *p < 0.1; **p < 0.05; ***p < 0.01

The event study figure in Fig. 2b shows that in the years following the mandatory-access PDMPs, there is again a delay of approximately 4 years, after which there is a sharp increase in the rate of heroin-involved IPV incidents in the medium run. The average treatment effects reported in the second column of Panel A of Table 1 imply that there is no evidence of a significant change in heroin-involved IPV rates immediately following the mandatory-access PDMP implementation, but the rate of heroin-involved IPV rate quadruples in the medium run.^10^ These findings are consistent with earlier studies showing that mandatory-access PDMPs can trigger substitution into illicit opioids including heroin, particularly for addicted individuals, as prescription opioids become more difficult to access (Meinhofer 2018; Kim 2021; Mallatt 2022). At the same time, the consumption of heroin has been documented to be strongly associated with a higher probability of IPV perpetration (El-Bassel et al. 2007; Tran et al. 2014). Nevertheless, heroin-related IPV represents a small proportion of all IPV incidents (less than 1%), and the notable increase in IPV incidents among the highly opioid-dependent individuals does not outweigh the overall decline in total IPV incidents in affected states.

**Fig. 3:**
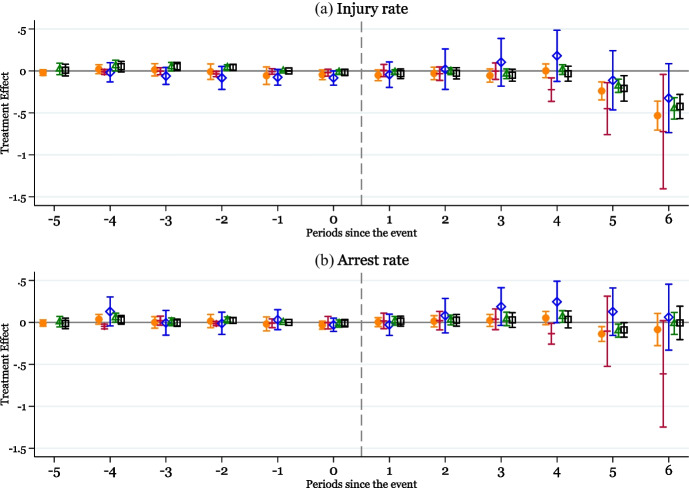
The effects of mandatory-access PDMPs on IPV rates over time. *Note:* Data are from the 2006–2019 NIBRS. Event-study plots show the response of injury rate and arrest rate per 1000 population reported by female victims at the county level (*N*=12,487 county-years) to mandatory-access PDMP implementation. This figure overlays the event-study plots based on Eq. (1) using five different estimators: Borusyak, Jaravel, and Spiess (2024) (in orange with circular markers); De Chaisemartin and d’Haultfoeuille (2020) (in red with cross markers); Callaway and Sant’Anna (2021) (in blue with diamond markers); Sun and Abraham (2021) (in green with triangle markers); and a dynamic version of the TWFE model, equation (1), and estimated using OLS (in blue with black square markers). Each figure reports treatment effect estimates and 95% confidence intervals. Specifications include county and year fixed effects, county-level covariates (percent female, White, Black, Hispanic population; number of cancer deaths per 100,000 population; percent population under age 19, between 20 and 24, between 25 and 34, between 35 and 44, between 45 and 54, and between 55 and 64; unemployment and labor force participation rates, the number of agencies reporting any IPV incidents), initial county characteristics (share of population without any college education and the share of employment in mining) interacted with year fixed effects, and state-level policies (indicators for a medical marijuana law and ACA expansion). Standard errors are clustered at the state level

Figure 3a shows that the event study results for the injury rate are consistent with those observed for the IPV incident rate. The average treatment effects reported in the first column of Panel B in Table 1 imply no evidence of a significant change in the injury rate in the short run but a 9.5% annual decline in the injury rate in the medium run. The similar medium-run decline in injuries related to IPV due to the implementation of mandatory-access PDMP laws implies that the overall decrease in reported IPV incidents to law enforcement agencies (Fig. 3a) is unlikely to be driven by a change in reporting behavior of the victims to the police, and more likely to represent a decline in actual incidents of IPV. In panel b, the event study estimates for the arrest rate are more noisily estimated, and while we see a downward shift for coefficients by some estimators, these effects are relatively small as can also be seen in column 2 of Panel B in Table 1.

**Table 2:** The effects of mandatory-access PDMPs on IPV sub-component rates

Panel A: Impact of mandatory-access PDMPs on IPV simple-assault rate and IPV aggravated-assault rate		
	Simple-assault IPV rate	Aggravated-assault IPV rate
	(1)	(2)
Short-run post-PDMP (0<t<3)	$-$0.0705	0.0088
	(0.0577)	(0.0211)
Medium-run post-PDMP (3<t<6)	$-$0.2429**	$-$0.0027
	(0.0980)	(0.0268)
Observations	12,487	12,487
Pre-policy outcome mean	2.0173	0.3471
Panel B: Impact of mandatory-access PDMPs on intimidation and forced sex rates		
	Intimidation rate	Forced sex rate
	(1)	(2)
Short-run post-PDMP (0<t<3)	0.0302*	0.0026
	(0.0177)	(0.0023)
Medium-run post-PDMP (3<t<6)	$-$0.0217	0.0077***
	(0.0253)	(0.0027)
Observations	12,487	12,487
Pre-policy outcome mean	0.3726	0.0611

*Notes:* Data are from the 2006–2019 NIBRS. Analyses show the response of IPV rate, heroin-involved IPV rate, injury rate, and arrest rate per 1000 population reported by female victims at the county level (*N*=12,487 county-years) to mandatory-access PDMP implementation. Estimates are calculated using the Borusyak et al. (2024) method using the specification in Eq. 2. Specifications include county and year fixed effects, county-level covariates (percent female, White, Black, Hispanic population; number of cancer deaths per 100,000 population; percent population under age 19, between 20 and 24, between 25 and 34, between 35 and 44, between 45 and 54, and between 55 and 64; unemployment and labor force participation rates, the number of agencies reporting any IPV incidents), initial county characteristics (share of population without any college education and the share of employment in mining) interacted with year fixed effects, and state-level policies (indicators for a medical marijuana law and ACA expansion). Standard errors in parentheses are clustered at the state level. *p < 0.1; **p < 0.05; ***p < 0.01

Table 2 further examines the effects of mandatory PDMPs on the sub-components of the IPV incidents. The results show that the reduction in IPV risk is driven primarily by the reduction in simple assaults, which make up the majority ($\sim$72%) of IPV incidents reported to law enforcement. We find no evidence of any significant effects on aggravated assault or intimidation rates in the medium run.^11^ However, we find that the introduction of mandatory PDMPs led to an increase in IPV incidents involving forced sex in the treated states. Though the point estimate is small, the effect size is statistically distinguishable from zero and translates to a 13% increase in the rate of such incidents (relative to the pre-intervention baseline mean of IPV involving forced sex). We note that forced sex comprises the smallest share of all reported IPV incidents ($\sim$2.2%), and this low prevalence makes it challenging to further dissect this outcome and explore this result further. However, it could be that the substitution from Rx opioids to heroin among a subset of users who saw their access to Rx opioids restricted may have led to a shift in the composition of IPV. Given our robust finding that mandatory PDMP provisions led to an increase in heroin-involved IPV in conjunction with the evolution of the opioid epidemic from Rx opioids to illicit opioids, it is possible that this substitution also resulted in a shift toward more serious IPV incidents, notably those involving forced sex.

### Heterogeneous treatment effects

In this section, we explore whether the effects of the mandatory-access PDMPs are heterogeneous by victim characteristics. To this end, we construct the incident rate for each population subgroup (i.e., non-Hispanic White/Black, Hispanic, younger/older than 30) and test whether some groups have experienced higher treatment effects than others. Specifically, for each subgroup, we first count the number of IPV incidents in a given county at a given year and then divide this count by the county population, which provides us with the rate of prevalence of IPV for a particular subgroup.^12^

**Fig. 4:**
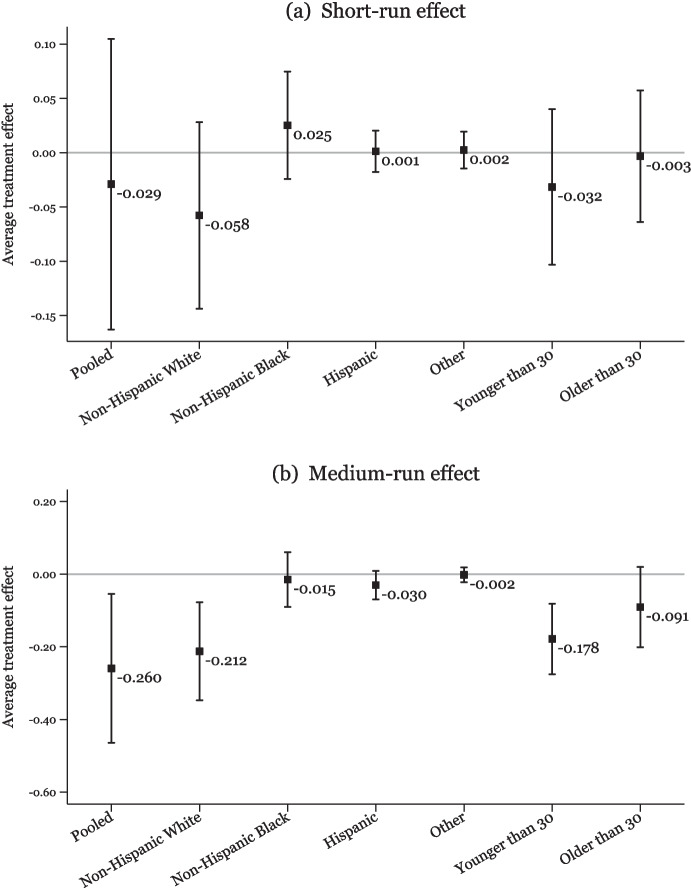
Heterogeneity by victim characteristics - IPV rate. *Note:* Data are from the 2006–2019 NIBRS. The figure shows heterogeneous treatment effects of mandatory-access PDMPs on the IPV rate per 1000 population by female victim’s characteristics. **a** reports short-run estimates covering the treatment period between 0 and 3 years after the PDMP implementation at the state level, and **b** reports estimates covering the treatment period between 4 and 6 years after the PDMP implementation at the state level as specified in Eq. 2. All estimates are calculated using the Borusyak et al. (2024) method. Vertical bars represent the 95% confidence intervals for these estimates

Figure 4 shows no evidence of a significant impact on any group in the short run (panel a), but in the medium run, the most substantial declines in IPV rates in response to mandatory-access PDMPs are observed among the non-Hispanic White population, with no evidence of a significant impact found for non-Hispanic Black, Hispanic, or other groups.^13^ Moreover, we also observe a larger reduction in IPV rates among younger adults; however, this decline is not statistically different than the decline for older adults. Overall, these results are consistent with the fact that prescription opioid consumption rates have historically been highest among non-Hispanic Whites and relatively younger population groups (Palmer et al. 2015; Humphreys et al. 2022). This aligns with our findings, as non-Hispanic Whites experience the strongest reductions in IPV following the policy intervention. Their higher baseline opioid consumption makes them more directly impacted by policies aimed at reducing opioid overprescription, resulting in more pronounced effects on IPV within this subgroup.

**Fig. 5:**
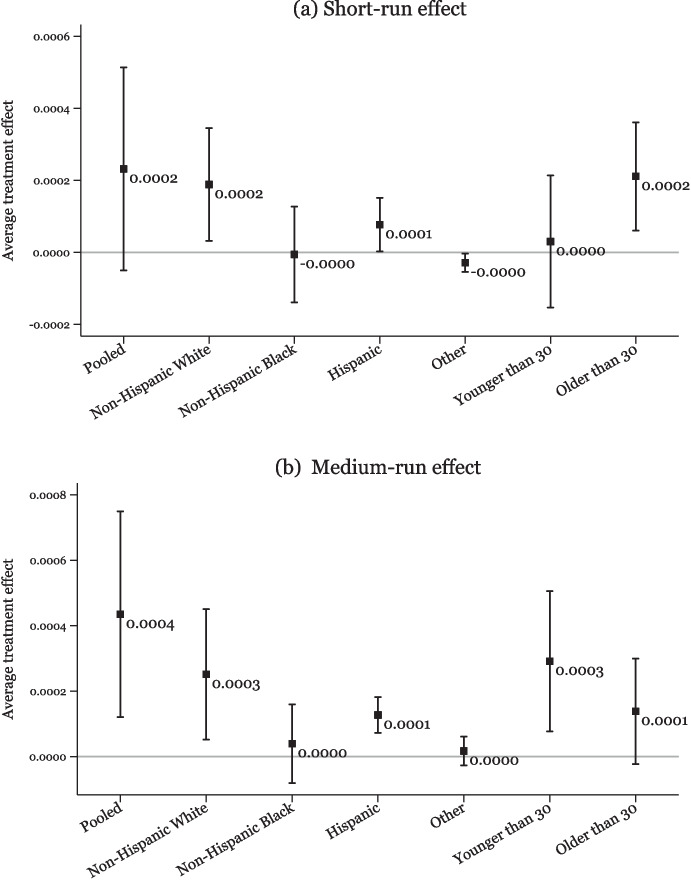
Heterogeneity by victim characteristics - heroin-involved IPV rate. *Note:* Data are from the 2006–2019 NIBRS. The figure shows heterogeneous treatment effects of mandatory-access PDMPs on the heroin-involved IPV rate per 1000 population by female victim’s characteristics. **a** reports short-run estimates covering the treatment period between 0 and 3 years after the PDMP implementation at the state level, and **b** reports estimates covering the treatment period between 4 and 6 years after the PDMP implementation at the state level as specified in Eq. 2. All estimates are calculated using the Borusyak et al. (2024) method. Vertical bars represent the 95% confidence intervals for these estimates

Figure 5 displays heterogeneous treatment effects of mandatory-access PDMPs on heroin-involved IPV rate. The results show the presence of a strong, positive treatment effect on non-Hispanic White population both in the short run and medium run after the policy change. We also see a smaller increase for the Hispanic group. The differences by age are not statistically different from each other. Additionally, Supplementary Material Figs. A1 and A2 indicate that the changes in injury and arrest rates in treated states exhibited similar patterns with respect to heterogeneous effects.

Furthermore, we also explore whether there is heterogeneity in treatment effects by educational attainment. While the NIBRS does not provide information on victim’s educational attainment or other markers of SES, we use data from the American Community Survey to calculate average college completion rates at the state level from 2005 to 2009. We then examine whether mandatory PDMPs differentially affect states above or below the median college completion rate (see Supplementary Material Fig. A3). The coefficient estimates for the medium-term impact of mandatory PDMPs on risk of IPV exposure are larger for states with below-median college completion rates, consistent with the fact that less educated populations were more affected by the first wave of the opioid epidemic. However, standard errors are not precise enough to reject the null that these estimates are statistically different from states with higher levels of educational attainment; hence, we interpret these patterns as suggestive rather than conclusive.

Finally, Supplementary Material Fig. A4 further shows that the decline in IPV rates in response to mandatory PDMPs is observed across all regions. However, these spillover effects are particularly strong in the Northeast and the West and imply decreases in the IPV rate of 22 and 18% (relative to the baseline mean), respectively; in comparison, while the South and Midwest also show significant decreases in IPV, these effects are relatively smaller (4 to 9%). These results are generally consistent with the relative intensity of the first wave of the opioid epidemic, with the Northeast and West being disproportionately impacted due to aggressive pharmaceutical marketing, high opioid prescribing rates, and a higher prevalence of non-Hispanic White populations, who were more vulnerable to opioid misuse (McGranahan and Parker 2021). Rural and suburban areas in these regions, facing chronic pain and labor-related injuries, experienced some of the highest rates of misuse and overdose mortality. States including New Hampshire, Vermont, and California were among the hardest hit. Our results thus suggest that the deployment of mandatory PDMPs generated the largest benefits in terms of IPV reductions in such regions with the highest prevalence of opioid misuse.

### Robustness checks

We examine the robustness of our findings by controlling for additional state policies that may impact opioid consumption and IPV incidence. Supplementary Material Table A3 presents our results by estimating Eq. 2. Specifically, we find that our results are robust to adding policy controls for the following: (i) Good Samaritan Laws, which provide legal immunity to individuals seeking assistance for someone during an overdose situation; (ii) Naloxone access laws, which increase the availability of Naloxone to people close to at-risk individuals, enabling them to administer it during an overdose; (iii) marijuana decriminalization policies, which reduce or eliminate criminal penalties for the possession and personal use of small amounts of marijuana, and recreational marijuana laws, which allow for recreational uses of marijuana, both of which might affect the tendency to substitute between opioids and marijuana; (iv) physical exam requirement (PER) laws, which require an in-person medical examination or a doctor-patient relationship before prescribing controlled substances; (v) the Earned Income Tax Credit (EITC) coverage, which varies by state within our sample period. Incorporating these additional policy controls resulted in estimates that were as precise, if not more precise, than those from our base specification presented in Table 1.

Moreover, we conduct additional sensitivity analyses to test whether the results are robust to different specification choices, covariates, and sample selection. Supplementary Material Table A4 presents the results. First, we cluster standard errors at the county level to account for serial correlation in outcomes within a county. Second, we control for the number of police officers per capita to account for differential changes in law enforcement capacity across counties. Finally, to ensure data quality when using incident data from NIBRS, we exclude counties with potentially insufficient IPV data reporting to further improve data quality for incidents reported in NIBRS.^14^ Our results remain robust to these alternative specifications and sample selections and consistent with those reported in Table 1.

Finally, we check whether voluntary PDMPs had any significant impact on IPV outcomes. Specifically, we include an indicator for having a PDMP of any form in Eq. 2 together with our mandatory-access PDMP indicators in short and medium run. Controlling for these mandatory-access PDMPs, the voluntary PDMPs’ impact can be seen in the estimates for any PDMP indicator. Supplementary Material Table A5 reports the results. These results show no evidence of a significant impact of having voluntary PDMPs, and our main coefficients of interest on mandatory-access PDMPs are entirely consistent with the ones in Table 1.

## Conclusion

As use and misuse of opioids surged over the past two decades, public health experts have expressed concerns regarding the role that opioid abuse can play in facilitating IPV (Warshaw et al. 2014; Packard and Warshaw 2018). There is very little causal evidence to date on the intersection of these two public health crises engendered by the rise in opioid use disorders and the high prevalence of IPV experienced by women, respectively. We address this knowledge gap and provide some of the first evidence on how supply-side interventions, in the form of prescription drug monitoring programs that restrict Rx opioid access for at-risk patients, are impacting women’s exposure to IPV. In the process, we also contribute to the nascent literature that recognizes that the opioid crisis has generated far-reaching consequences on non-users, families, and communities and has widened the lens to evaluate potential spillover effects of opioid policies on a broader range of health, economic, and social outcomes.

Capitalizing on administrative data on incidents reported by female victims to law enforcement, in conjunction with quasi-experimental variation in the adoption of stringent must-access PDMP provisions, we find that these policies have generated a downstream benefit for women’s health by significantly reducing their overall exposure to IPV and IPV-involved injuries by 9 to 10%. Strongest effects are experienced by non-Hispanic whites and younger adults, which is validating given that these groups have among the highest rates of opioid abuse and have been found to display relatively larger first-stage responses.^15^

Our findings are in line with Dave et al. (2021), who study effects on criminal activity more broadly and find decreases in certain forms of violent offenses (assault) and property crimes (burglary and motor vehicle theft). The results from our study confirm that the lower rates of violent crime perpetration also extend to intimate partners and confer important gains for women’s well-being. In studying criminal activity, Dave et al. (2021) caution, however, that their finding of a net decline in overall crime does not preclude an increase in criminal engagement for a subset of individuals due to substitution into heroin or alternate illicit sources of opioid supply. In the context of IPV perpetration, we find support for such an underlying substitution response. Specifically, while our main results point to overall reduced IPV exposure for women, our analyses identify a significant increase in IPV incident reports where the perpetrator is suspected of using heroin. This uptick in heroin-involved IPV is not nearly large enough to fully offset the lower overall risk of IPV exposure. Nevertheless, this result underscores an important unintended consequence identified in the literature wherein some users of Rx opioids are substituting into heroin as their access to the former is being constrained (Alpert et al. 2018; Evans et al. 2019; Kim 2021; Alpert et al. 2022; Mallatt 2022; Dave et al. 2023). Such substitution effects translate into a greater incidence of IPV perpetration against women committed by impacted users, implying that the gains to health and well-being that we find are not experienced uniformly by all impacted women.

Despite documented associations between substance abuse and IPV, the causal role played by Rx opioid misuse in driving IPV perpetration/victimization remains unclear. We can shed light on this causal role by combining our reduced-form effects on IPV with those on Rx opioid misuse from the literature to impute an “ballpark” structural elasticity of IPV with respect to Rx opioid misuse. To do so, we utilize estimates of the latter from Grecu et al. (2019) who find that mandatory PDMPs were effective in reducing opioid misuse among young adults by between 26 and 34% (26% for Rx opioid overdose mortality and 34% for opioid-related treatment admission flows) in conjunction with our estimated effect indicating a 9.3% decrease in IPV perpetrated against women. This implies a structural elasticity of IPV with respect to Rx opioid misuse on the order of 0.27 to 0.36.^16^ By applying these elasticity estimates to the baseline number of adults misusing opioids and IPV incidents against women, we can derive a marginal IPV response among deterred opioid abusers of approximately 0.02. In other words, for every 50 or so fewer Rx opioid abusers as a result of constraining their access to opioids (due to PDMPs), about one female victim-reported IPV incident appears to have been averted on the margin.^17^^,^^18^ The annual economic toll of female-experienced IPV is staggering, amounting to $\$$11.6 billion (in 2019 dollars) (Max et al. 2004). Our estimates suggest that approximately 9% of these costs, or $\$$1.04 billion annually, can be attributed to opioid-driven IPV incidents, adding to the societal burden imposed by the opioid crisis each year.^19^

## Supplementary Information

Below is the link to the electronic supplementary material.

Supplementary file 1 (pdf 149 KB)

## Data Availability

Program files to replicate the results presented in the paper are available on request from the corresponding author.

## Footnotes

1 The authors’ computation of overdose deaths involving any opioid (ICD-10 multiple cause of death codes: T40.1, heroin; T40.2, natural/semi-synthetic opioids; T40.3, methadone; T40.4, other synthetic opioids), between 1999 and 2022, derived from CDC WONDER (https://wonder.cdc.gov/).

2 See Florence et al. (2021) and the report of the Joint Economic Committee (2022); latter available at https://www.jec.senate.gov/public/index.cfm/democrats/2022/9/the-economic-toll-of-the-opioid-crisis-reached-nearly-1-5-trillion-in-2020.

3 The CDC study (Florence et al. 2021), for instance, underscores that its cost estimates are conservative since they do not reflect several additional costs, including those imposed on non-users (i.e., infants; neonatal abstinence syndrome) or costs of all other criminal activity (beyond just drug-related offenses) associated with opioids.

4 Experiencing abuse by an intimate partner can adversely impact the physical and mental health of women (including injuries, chronic pain, depression, anxiety, other trauma-related mental health conditions, unwanted pregnancies, and sexually transmitted diseases, death) both in the short term and the long term (Campbell 2002; World Health Organization 2013) and have adverse developmental consequences for children who witness IPV (Stiller et al. 2022; Wood and Sommers 2011). Among homicides where the perpetrator is known, over one-half of female victims are killed by their current or former intimate partner (Ertl et al. 2019).

5 In a study of 484 drugs associated with serious adverse events reported to the FDA, Moore et al. (2010) find that oxycodone (one of the higher-potency opioids, and the most commonly prescribed opioid in the U.S. by 2010) was among the top 20 drugs that showed a disproportionate association with violence towards others. Studies have also shown that opioid-dependent fathers exhibited more violent and aggressive behaviors towards their intimate partners (Moore et al. 2011).

6 See, for instance, Castilla and Murphy (2023), Chalfin et al. (2021), Angelucci and Heath (2020), and Markowitz (2005). Using a randomized control trial that mitigates alcohol consumption in rural Kenya, Castilla et al. (2022) find that reductions in alcohol use substantially lower sexual violence, though not other forms of physical or emotional violence, perpetrated towards intimate partners. Evidence on the interplay between opioids and IPV perpetration/victimization has been limited to the co-occurrence of opioid use/misuse and IPV (Hughes et al. 2019; Jessell et al. 2017; Stone and Rothman 2019).

7 See Max et al. (2004) and Peterson et al. (2018).

8 The United States comprises 50 states, each with an average of approximately 62 counties. In total, there are 3142 counties across the country (U.S. Census Bureau 2025).

9 This decline implies a $-$0.2596 percentage point reduction as a share of the pre-policy outcome mean of 2.7980 ($-$0.2596/2.7980*100).

10 Information on whether the IPV offender is suspected of using heroin (or other substances) is derived from law enforcement personnel’s perceptions and observations and/or victim reports. Hence, involvement of heroin in the IPV incident is measured with noise, and likely under-reported. To the extent that this measurement error is orthogonal to the timing of PDMP regulations, the relative policy effects should remain unbiased. Nevertheless, these results should be interpreted as suggestive rather than conclusive in light of the imperfect measures of substance involvement in the NIBRS data.

11 We observe a very small and marginally significant increase in intimidation rate in the short run; however, this effect turns negative in the medium run. We observe no significant effects in aggravated-assault rate in the short run or medium run.

12 For this reason, the pooled estimate corresponds to the sum of coefficients for each subgroup, instead of its weighted average.

13 We acknowledge that reporting biases could influence our results, particularly for Hispanic and non-White subgroups, as previous research has noted that these populations may underreport IPV incidents to law enforcement due to factors such as mistrust in authorities, fear of deportation, or cultural stigmas (Vidales 2010; Erez et al. 2009). While these biases may lead to an underestimation of IPV incidents for these groups, we expect them to be consistent over time and across states, minimizing their impact on our estimates of policy effects.

14 Following Fone et al. (2023), we use 65% coverage rate. However, the estimates are quite similar across different cutoffs. The coverage indicator is the effective coverage of reporting of IPV by agencies for a given county and year. If the indicator approaches 100, it signifies near-complete coverage, meaning the agencies report data for the entire year and encompass the entire population. Conversely, if the indicator is near 0, the coverage is minimal, indicating the agencies report data for only a small portion of the year or cover a small segment of the population. Following Fone et al. (2023), we calculate the coverage indicator by using the following expression:

3 $$\begin{aligned} CI_{c,t} = (1 - \sum _{a=1}^{n_{c,t}} \left( \frac{A_{a,c,t}}{T_{c,t}} \cdot \frac{12 - M_{a,t}}{12} \right) ) \times 100 \end{aligned}$$

where $CI_{c,t}$ is the coverage indicator for county *c* in year *t*; $n_{c,t}$ is the number of agencies in county *c* at time *t*; $A_{a,c,t}$ is the population of agency *a* in county *c* in year *t*; $T_{c,t}$ is the total population in county *c* in year *t*; and $M_{a,t}$ is the number of months agency *a* reported in year *t*.

15 Grecu et al. (2019), for instance, find that opioid misuse and mortality among younger adults (relative to older adults) are most responsive to mandatory-access PDMPs, which would then be expected to translate into relatively larger downstream effects, ceteris paribus.

16 Imputing structural “treatment on the treated” elasticity estimates in this way is a noisy endeavor, and these estimates are meant to be suggestive rather than definitive. Small changes in the underlying reduced-form parameters can lead to large changes in the imputed estimates. Moreover, the two reduced-form estimates (on IPV from our study and on opioid misuse from Grecu et al. 2019) do not perfectly align with respect to samples and empirical specifications. Nonetheless, that these imputed marginal responses are largely in line with the average prevalence of IPV perpetration among males who misuse opioids (approximately 20 to 38%; see Stone and Rothman 2019) instills a degree of confidence that the effect sizes we find are plausible.

17 The 2006 National Survey of Drug Use and Health (NSDUH) reported 4.529 million adults had misused pain relievers in the past month, and our analyses with the NIBRS indicate 317,581 female victim-reported IPV incidents.

18 With the annual and lifetime economic burden of IPV against women estimated at $\$$11.6 billion and $\$$3.65 trillion respectively (in 2019$\$$; see: Max et al. 2004 and Peterson et al. 2018)., implied net cost savings realized from an overall decline in female-experienced IPV associated with the deployment of mandatory-access PDMPs nationally can amount to $\$$1.04 billion annually and $\$$328.5 billion over the lifetime of female IPV victims. It is important to account for such cost savings realized through broader unintended impacts, as well as potential distributional impacts (as we find in this study, with some subsets of women experiencing an increase in harms due to greater exposure to heroin-involved IPV) for effective policy guidance and for identifying sub-populations who may experience unintended harms.

19 For this exercise, we utilize the total number of adults in the 2019 NSDUH who reported misusing Rx pain relievers in the past month (1.333 million) and the total number of IPV incidents from the 2019 NIBRS (304,000), in conjunction with the imputed marginal IPV response (0.02), in order to apportion the fraction of IPV cases with female victims that can be attributed to opioid misuse: [(1.333 million $\times$ 0.02)/304,000 = 0.09].
