# Supplementary material for: Fighting abuse with prescription tracking: mandatory drug monitoring and intimate partner violence
Source: J Popul Econ. 2025 Jun 28;38(3):57. doi: 10.1007/s00148-025-01111-5 (PMC12206206; doi:10.1007/s00148-025-01111-5)
Supplement: Supplementary file 1 — (pdf 149 KB) [file 148_2025_1111_MOESM1_ESM.pdf]

APPENDIX

Figure A1: Heterogeneity by Victim Characteristics - Injury Rate

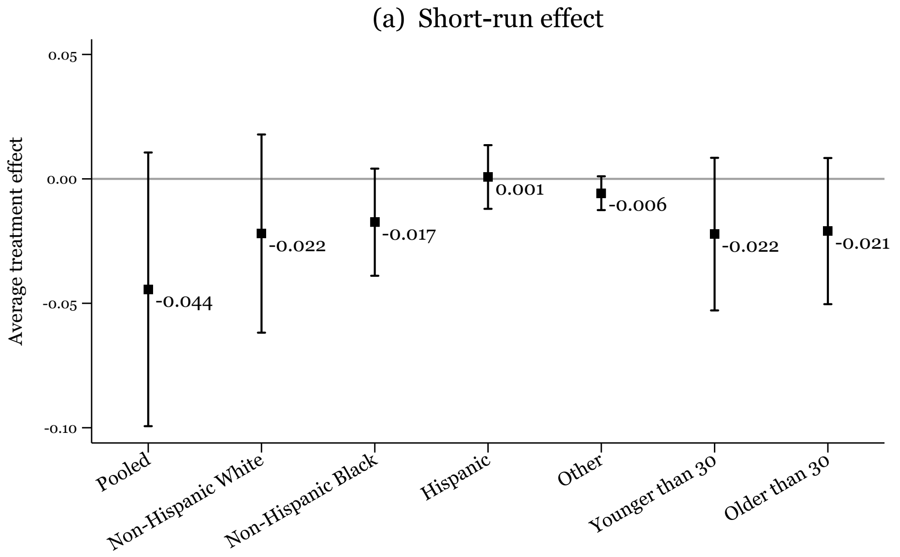

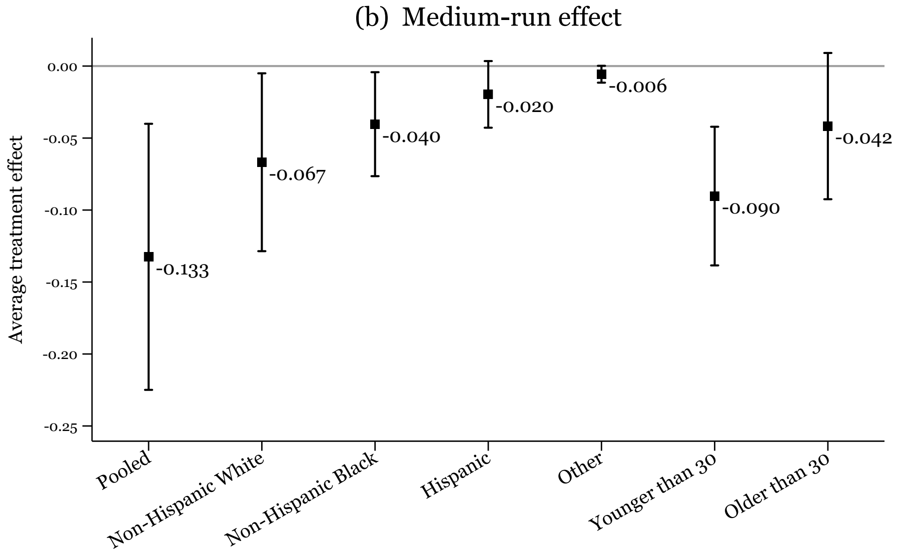

Note: Data are from the 2006–2019 NIBRS. The figure shows heterogeneous treatment effects of mandatory-access PDMPs on the injury rate per 1,000 population by female victim's characteristics. Panel (a) reports short-run estimates covering the treatment period between 0 and 3 years after the PDMP implementation at the state level, and Panel (b) reports estimates covering the treatment period between 4 and 6 years after the PDMP implementation at the state level as specified in equation 2. All estimates are calculated using the Borusyak et al. (2024) method. Vertical bars represent the 95% confidence intervals for these estimates.

Figure A2: Heterogeneity by Victim Characteristics - Arrest Rate

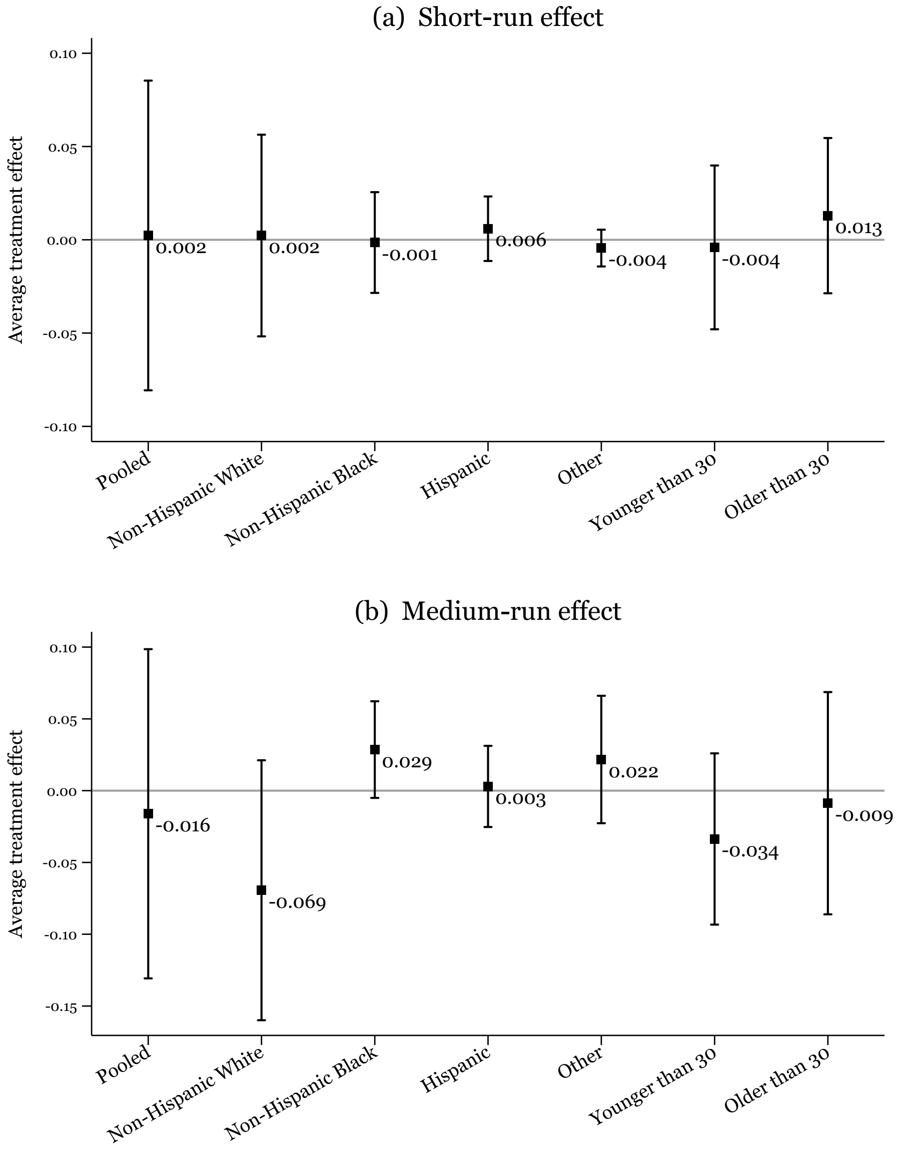

Note: Data are from the 2006–2019 NIBRS. The figure shows heterogeneous treatment effects of mandatory-access PDMPs on the arrest rate per 1,000 population by female victim's characteristics. Panel (a) reports short-run estimates covering the treatment period between 0 and 3 years after the PDMP implementation at the state level, and Panel (b) reports estimates covering the treatment period between 4 and 6 years after the PDMP implementation at the state level as specified in equation 2. All estimates are calculated using the Borusyak et al. (2024) method. Vertical bars represent the 95% confidence intervals for these estimates.

Figure A3: Heterogeneity by College Completion Rate

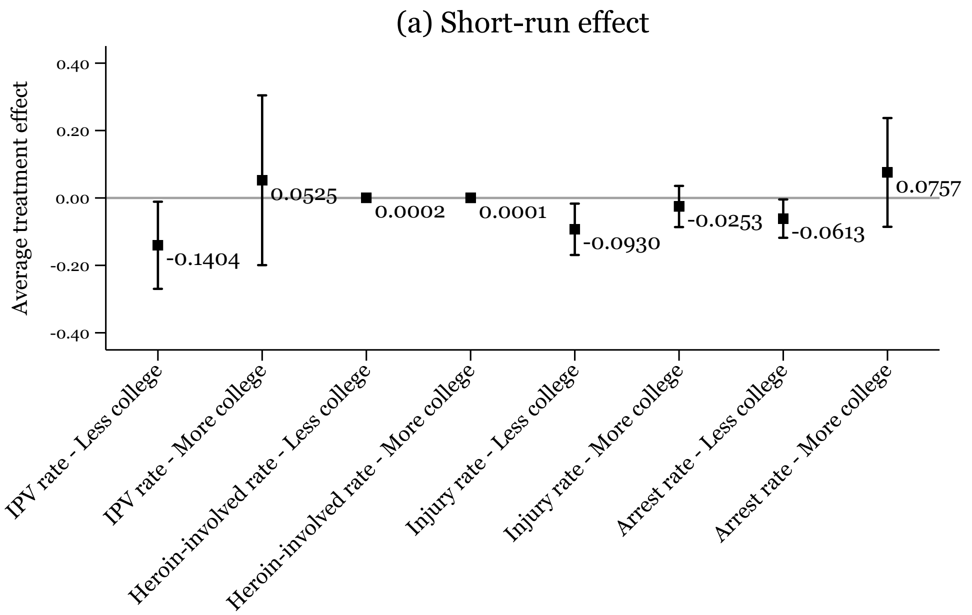

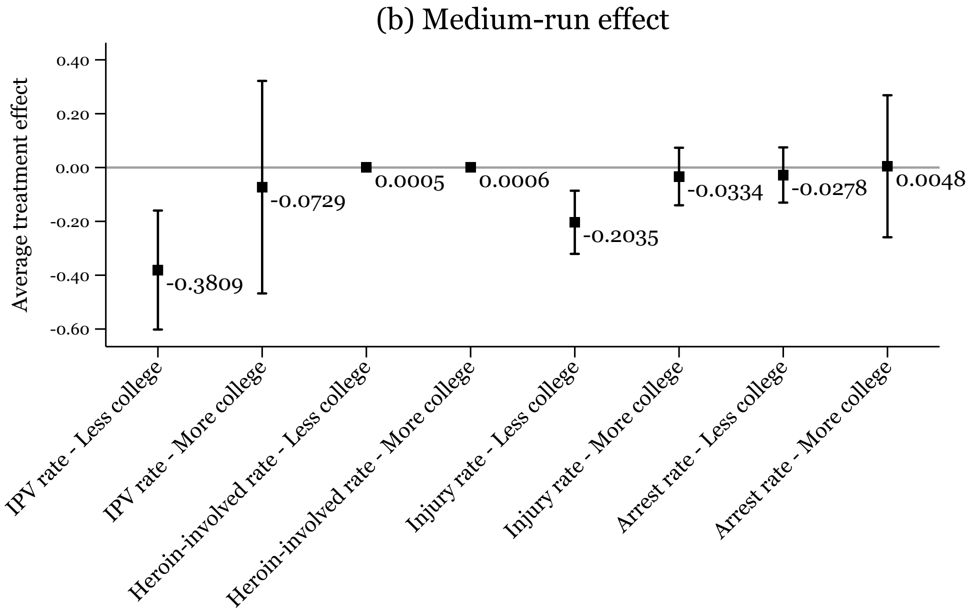

Note: Data are from the 2006–2019 NIBRS. The figure shows heterogeneous treatment effects of mandatory-access PDMPs on IPV rate, heroin-involved IPV rate, injury rate, and arrest rate per 1,000, by whether a state's college-completion rate is above or below the median over the period 2005-2009. Panel (a) reports short-run estimates covering the treatment period between 0 and 3 years after the PDMP implementation at the state level, and Panel (b) reports estimates covering the treatment period between 4 and 6 years after the PDMP implementation at the state level as specified in equation 2. All estimates are calculated using the Borusyak et al. (2024) method. Vertical bars represent the 95% confidence intervals for these estimates.

Figure A4: Heterogeneity by Regions - IPV Rate

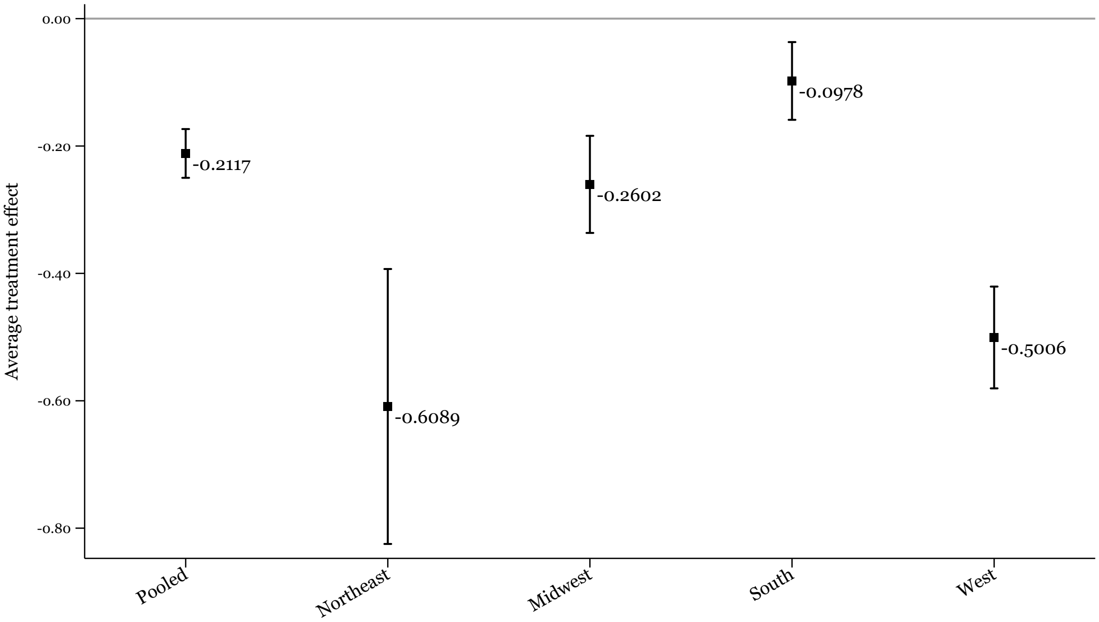

Note: Data are from the 2006–2019 NIBRS. The figure shows the heterogeneous treatment effects of mandatory-access PDMPs on the IPV rate per 1,000 people based on the regions in which it occurred. Estimates are obtained using a Two-Way Fixed Effects regression. The pooled coefficients represent the estimates for the full sample, and separate estimates are shown for regional subsamples, including the Northeast, Midwest, South, and West. Vertical bars represent the 95% confidence intervals for these estimates.

Table A1: Mandatory-Access PDMP Implementation Years by State

Year	States				
2007	NV				
2008					
2009					
2010					
2011	OH	MA			
2012	DE	KY	NM	WV	
2013	NY	TN			
2014	IN	LA			
2015	VT	CT	NJ	VA	OK
2016	NH	RI			
2017	PA				

Notes: Must-access PDMP implementation dates were taken from Evans et al. (2022), but with three corrections and one addition.

1. Oklahoma Code § 535:15-3-9 is the statute was first enacted in 2010 for methadone. It was expanded to the relevant controlled substances in 2015.

2. Vermont 18 V.S.A. § 4289 was amended 2015, No. 173 (Adj. Sess.), § 2.

3. Massachusetts statute 247 Mass. Reg. 5.04 became effective January 1, 2011.

4. Pennsylvania "Amended by P.L. TBD 2016 No. 124, § 3, eff. 1/1/2017." 35 Pa. Stat. § 872.7

Table A2: Summary Statistics

	Mean	SD	Min	Max	N
Intimate partner violence rate (per 1,000)	2.62	1.90	0.00	15.07	12487
Heroin-involved intimate partner violence rate (per 1,000)	0.00	0.00	0.00	0.15	12487
Injury rate (per 1,000)	1.33	0.96	0.00	9.09	12487
Arrest rate (per 1,000)	1.44	0.98	0.00	7.98	12487
Indicator for MA PDMP	0.20	0.40	0.00	1.00	12487
Percent Black	0.08	0.12	0.00	0.74	12487
Percent White	0.89	0.13	0.19	1.00	12487
Percent Hispanic	0.06	0.08	0.00	0.64	12487
Percent under age 0 to 19	0.25	0.03	0.12	0.40	12487
Percent age 20 to 24	0.06	0.03	0.02	0.28	12487
Percent age 25 to 34	0.12	0.02	0.05	0.28	12487
Percent age 35 to 44	0.12	0.02	0.06	0.20	12487
Percent age 45 to 54	0.14	0.02	0.06	0.22	12487
Percent age 55 to 64	0.14	0.02	0.05	0.25	12487
Percent age over age 64	0.17	0.04	0.04	0.38	12487
Cancer deaths per 100,000 population	237.10	68.97	35.26	697.67	12487
Unemployment rate	6.06	2.97	1.10	25.60	12487
Labor force participation rate	0.60	0.08	0.28	1.27	12487
Indicator for any PDMP	0.89	0.31	0.00	1.00	12487
Percent without any college education	0.48	0.11	0.13	0.80	12487
Percent employment in mining	0.01	0.03	0.00	0.28	12487
Indicator for having medical marijuana law	0.28	0.45	0.00	1.00	12487
Number of agencies reporting any IPV incidents	3.93	4.53	1.00	56.00	12487
Indicator for ACA expansion	0.24	0.42	0.00	1.00	12487

Notes: The table presents the means, standard deviations, minimum and maximum values, and the number of observations for variables used in the analysis at the county level from 2006–2019 NIBRS (N=12,487 county-years).

Table A3: Robustness Analysis-I

	IPV rate per 1,000 population	Heroin-involved IPV rate per 1,000 population	Injury rate per 1,000 population	Arrest rate per 1,000 population
Controlling for the following policies:				
Good Samaritan Laws				
Short-run post-PDMP (0≤t≤3)	-0.0249	0.0002	-0.0391	0.0050
	(0.0914)	(0.0002)	(0.0375)	(0.0523)
Medium-run post-PDMP (3<t≤6)	-0.4892***	0.0006***	-0.2200***	-0.0985
	(0.1639)	(0.0002)	(0.0778)	(0.0852)
Naloxone Laws				
Short-run post-PDMP (0≤t≤3)	-0.0282	0.0002	-0.0520	-0.0059
	(0.0887)	(0.0002)	(0.0383)	(0.0521)
Medium-run post-PDMP (3<t≤6)	-0.4907***	0.0007***	-0.2320***	-0.1110
	(0.1721)	(0.0002)	(0.0847)	(0.0900)
Decriminalization of Marijuana				
Short-run post-PDMP (0≤t≤3)	-0.0245	0.0002	-0.0384	0.0013
	(0.0764)	(0.0002)	(0.0312)	(0.0485)
Medium-run post-PDMP (3<t≤6)	-0.2518**	0.0004**	-0.1220**	-0.0179
	(0.1176)	(0.0002)	(0.0541)	(0.0659)
Recreational Marijuana Laws				
Short-run post-PDMP (0≤t≤3)	-0.0394	0.0002	-0.0497	-0.0011
	(0.0801)	(0.0002)	(0.0345)	(0.0485)
Medium-run post-PDMP (3<t≤6)	-0.5159***	0.0007***	-0.2322***	-0.1130
	(0.1692)	(0.0002)	(0.0825)	(0.0880)
Physical Examination Requirements				
Short-run post-PDMP (0≤t≤3)	-0.0337	0.0003**	-0.0371	-0.0001
	(0.0775)	(0.0001)	(0.0345)	(0.0425)
Medium-run post-PDMP (3<t≤6)	-0.5815***	0.0007***	-0.2890***	-0.1853**
	(0.1794)	(0.0002)	(0.0921)	(0.0906)
EITC Policy				
Short-run post-PDMP (0≤t≤3)	-0.0247	0.0002	-0.0414	0.0049
	(0.0812)	(0.0002)	(0.0338)	(0.0497)
Medium-run post-PDMP (3<t≤6)	-0.2497**	0.0004**	-0.1257**	-0.0102
	(0.1241)	(0.0002)	(0.0559)	(0.0679)

Notes: Data are from the 2006–2019 NIBRS. The table shows the response of IPV rate, heroin-involved IPV rate, injury rate, and arrest rate per 1,000 population reported by female victims at the county level (N=12,487 county-years) to mandatory-access PDMP implementation. Estimates are calculated using the Borusyak et al. (2024) method using the specification in equation (2). Estimates are calculated using the Borusyak et al. (2024) method using the specification in equation (2). Specifications include county and year fixed effects, county-level covariates (percent female, White, Black, Hispanic population; number of cancer deaths per 100,000 population; percent population under age 19, between 20 and 24, between 25 and 34, between 35 and 44, between 45 and 54, and between 55 and 64; unemployment and labor force participation rates, the number of agencies reporting any IPV incidents), initial county characteristics (share of population without any college education and the share of employment in mining), and state-level policies (indicators for a medical marijuana law and ACA expansion). Standard errors in parentheses are clustered at the state level. ***, **, and * denote significance at the 1, 5, and 10 percent levels.

Table A4: Robustness Analysis-II

	IPV rate per 1,000 population	Heroin-involved IPV rate per 1,000 population	Injury rate per 1,000 population	Arrest rate per 1,000 population
Clustering at the county level				
Short-run post-PDMP (0≤t≤3)	-0.0290	0.0002	-0.0444	0.0023
	(0.0880)	(0.0002)	(0.0489)	(0.0408)
Medium-run post-PDMP (3<t≤6)	-0.2596*	0.0004*	-0.1325*	-0.0161
	(0.1477)	(0.0003)	(0.0694)	(0.0604)
Observations	12487	12487	12487	12487
Controlling for police per capita (in logs)				
Short-run post-PDMP (0≤t≤3)	-0.0240	0.0002	-0.0381	0.0071
	(0.0820)	(0.0002)	(0.0324)	(0.0492)
Medium-run post-PDMP (3<t≤6)	-0.2529**	0.0004**	-0.1240**	-0.0096
	(0.1259)	(0.0002)	(0.0576)	(0.0708)
Observations	12487	12487	12487	12487
Dropping counties below 65% coverage rate				
Short-run post-PDMP (0≤t≤3)	-0.0149	0.0002	-0.0394	0.0091
	(0.0847)	(0.0002)	(0.0345)	(0.0534)
Medium-run post-PDMP (3<t≤6)	-0.2596**	0.0005***	-0.1457**	-0.0177
	(0.1272)	(0.0002)	(0.0581)	(0.0722)
Observations	9010	9010	9010	9010

Notes: Data are from the 2006–2019 NIBRS. The table shows the response of IPV rate, heroin-involved IPV rate, injury rate, and arrest rate per 1,000 population reported by female victims at the county level. County-year observations are noted for each regression. Estimates are calculated using the Borusyak et al. (2024) method using the specification in equation (2). Estimates are calculated using the Borusyak et al. (2024) method using the specification in equation (2). Specifications include county and year fixed effects, county-level covariates (percent female, White, Black, Hispanic population; number of cancer deaths per 100,000 population; percent population under age 19, between 20 and 24, between 25 and 34, between 35 and 44, between 45 and 54, and between 55 and 64; unemployment and labor force participation rates, the number of agencies reporting any IPV incidents), initial county characteristics (share of population without any college education and the share of employment in mining), and state-level policies (indicators for a medical marijuana law and ACA expansion). Standard errors in parentheses are clustered at the state level. ***, **, and * denote significance at the 1, 5, and 10 percent levels.

Table A5: The Effects of Mandatory-Access PDMPs on IPV Rates Controlling for Having Any PDMP

Panel A: Impact of mandatory-access PDMPs on IPV rate and heroin-involved IPV rate		
	IPV rate per 1,000 population (1)	Heroin-involved IPV rate per 1,000 population (2)
Short-run post-PDMP (0<t<3)	-0.0465	0.0003*
	(0.0812)	(0.0002)
Medium-run post-PDMP (3<t<6))	-0.2818**	0.0005***
	(0.1223)	(0.0002)
Any PDMP	-0.1136	0.0002
	(0.1692)	(0.0002)
Observations	12,487	12,487
Pre-policy outcome mean	2.7980	0.0001

Panel B: Impact of mandatory-access PDMPs on injury and arrest rates		
	Injury rate per 1,000 population (1)	Arrest rate per per 1,000 population (2)
Short-run post-PDMP (0<t<3)	-0.0451	-0.0050
	(0.0359)	(0.0463)
Medium-run post-PDMP (3<t<6)	-0.1334**	-0.0253
	(0.0594)	(0.0636)
Any PDMP	-0.0046	-0.0472
	(0.0727)	(0.0854)
Observations	12,487	12,487
Pre-policy outcome mean	1.4014	1.4988

Notes: Data are from the 2006–2019 NIBRS. The table shows the response of IPV rate, heroin-involved IPV rate, injury rate, and arrest rate per 1,000 population reported by female victims at the county level (N=12,487 county-years) to mandatory-access PDMP implementation. Estimates are calculated using the Borusyak et al. (2024) method using the specification in equation (2), controlling for an indicator for having a PDMP of any form. Estimates are calculated using the Borusyak et al. (2024) method using the specification in equation (2). Specifications include county and year fixed effects, county-level covariates (percent female, White, Black, Hispanic population; number of cancer deaths per 100,000 population; percent population under age 19, between 20 and 24, between 25 and 34, between 35 and 44, between 45 and 54, and between 55 and 64; unemployment and labor force participation rates, the number of agencies reporting any IPV incidents), initial county characteristics (share of population without any college education and the share of employment in mining), and state-level policies (indicators for a medical marijuana law and ACA expansion). Standard errors in parentheses are clustered at the state level. ***, **, and * denote significance at the 1, 5, and 10 percent levels.

References

Borusyak, Kirill, Xavier Jaravel, and Jann Spiess, "Revisiting event-study designs: robust and efficient estimation," *Review of Economic Studies*, 2024, p. rdae007.
